# Reconciling Deep Calibration and Demographic History: Bayesian Inference of Post Glacial Colonization Patterns in *Carcinus aestuarii* (Nardo, 1847) and *C. maenas* (Linnaeus, 1758)

**DOI:** 10.1371/journal.pone.0028567

**Published:** 2011-12-02

**Authors:** Ilaria A. M. Marino, Jose Martin Pujolar, Lorenzo Zane

**Affiliations:** Department of Biology, University of Padova, Padova, Italy; Biodiversity Insitute of Ontario - University of Guelph, Canada

## Abstract

A precise inference of past demographic histories including dating of demographic events using Bayesian methods can only be achieved with the use of appropriate molecular rates and evolutionary models. Using a set of 596 mitochondrial cytochrome c oxidase I (COI) sequences of two sister species of European green crabs of the genus *Carcinus* (*C. maenas* and *C. aestuarii*), our study shows how chronologies of past evolutionary events change significantly with the application of revised molecular rates that incorporate biogeographic events for calibration and appropriate demographic priors. A clear signal of demographic expansion was found for both species, dated between 10,000 and 20,000 years ago, which places the expansions events in a time frame following the Last Glacial Maximum (LGM). In the case of *C. aestuarii*, a population expansion was only inferred for the Adriatic-Ionian, suggestive of a colonization event following the flooding of the Adriatic Sea (18,000 years ago). For *C. maenas*, the demographic expansion inferred for the continental populations of West and North Europe might result from a northward recolonization from a southern refugium when the ice sheet retreated after the LGM. Collectively, our results highlight the importance of using adequate calibrations and demographic priors in order to avoid considerable overestimates of evolutionary time scales.

## Introduction

Molecular data are the sole font of information to infer the demographic history of a species when a complete fossil record is lacking. The molecular clock has become a fundamental tool within evolutionary biology, facilitating independent time-scales to be placed on evolutionary events, such as glaciations and sea level changes [1]–[3]. Yet molecular chronologies crucially depend on accurate estimates of substitution rates and on the use of an appropriate evolutionary model. The initial assumption of a constant rate of evolution among lineages was challenged by results from datasets showing considerable departures from clockwise evolution [4]–[6]. Such problems with the molecular clock hypothesis have been partially addressed by the introduction in the past two decades of a number of relaxed-clock models, which allow for rate variation between and within lineages in a phylogeny [7]–[11]. On this subject, the advent of Bayesian methods provides the means to explore the demographic history of a species/genus, to use flexible models of population size changes through time (i.e. Bayesian skyline plots) and to take into account the effects of demography on the overall pattern of genetic variation [12], [13]. Moreover, the use of calibration points makes it possible to directly incorporate into the analysis information from fossils, biogeographic events and/or radio-carbon dated sequences, as well as to take into account uncertainty in calibration dates [9], [14].

Although it has long been recognized that different genes and taxa experience different rates of DNA change, recent studies have yielded a remarkable disparity in molecular rates estimated from population-level and pedigree data (mutation rate) compared to phylogenetic studies (substitution rate) [15], which can differ by up to an order of magnitude [16]. The time dependency hypothesis argues for an inconstant molecular clock over time to explain such rate differences [15], [17], [18]. A direct consequence of the time dependency of molecular rate estimates is that recent divergence times could be biased when using deep fossil calibration, resulting in divergence estimates older than they actually are. The extent of this bias is still under debate [19]–[24].

Ho *et al.* [25] emphasized the need for appropriate calibration, especially when deep fossil calibrations or universal substitution rates (e.g., 1% per million years) inferred from inter-specific comparisons are used on studies at the intraspecific level. The extrapolation of rates across the population-species boundary can yield invalid estimates of molecular time-scales, with significant impacts on subsequent evolutionary and ecological inferences [25]–[27]. When assessing the effect of inappropriate calibration using several case studies (avian speciation in the Late Pleistocene, the demographic history of bowhead whales, and the Pleistocene biogeography of brown bears), Ho *et al.* [25] showed that confoclusions changed significantly when applying revised internally-calibrated substitution rates.

**Figure 1:**
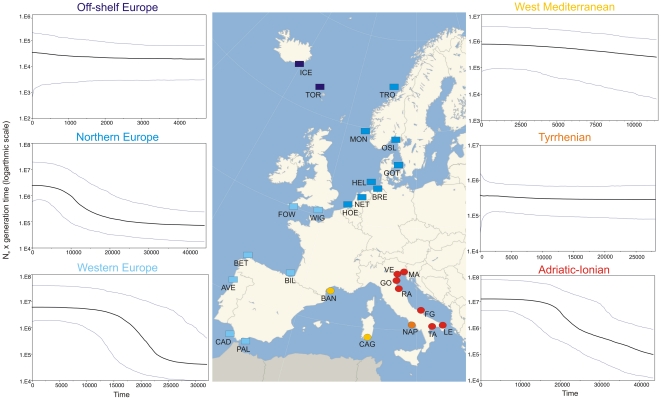
Distribution of collection sites and Bayesian skyline plot. Sampling locations of *Carcinus* genus in Europe and demographic histories of all *C. aestuarii* and *C. maenas* groups using Bayesian skyline plots are shown. Reported are the plots of *N*_e_ (effective population size multiplied per generation time) against time in years from present. Mean *N*_e_ is shown as a solid black line and light blue lines enclose the 95% highest posterior density interval. Samples are labelled as in Table 1. The map shows the groups identified by AMOVA and MDS analyses for *C. aestuarii* (circles) and *C. maenas* (squares), coded in different colours.

Here we illustrate the calibration problem using the case study of the evolutionary history of the green crabs of the genus *Carcinus*, which consists of a sibling species pair with an allopatric native distribution, *C. maenas* in the North-West Atlantic Ocean and *C. aestuarii* in the Mediterranean Sea (Fig. 1). Some particular life-history traits of the species (wide environmental tolerance including euryhalinity, high fecundities, long larval stages) make them successful invasive species, particularly *C. maenas*, which has colonized littoral habitats around the world [28], [29]. The taxonomic status of the Atlantic and Mediterranean forms was initially debated based on a lack of distinction in the larval stages [30] and in early allozyme data [31]. However, despite substantial overlap in morphometric and ecological characteristics, the adult stages show morphological diagnostic characters that indicate a species split between the forms of the Atlantic and Mediterranean basins [32]. The species status was proposed by Demeusy [33], who described the Mediterranean *Carcinus* as a Tethys remnant and proposed that the complete isolation of Atlantic and Mediterranean during the Messinian Salinity Crisis (5.59–5.33 MY ago; [34]) promoted allopatric speciation in the genus. This view is supported by the current understanding that estuaries functioned during the whole range of the Messinian Salinity Crisis, when shallow marginal water bodies with brackish, marine to hypersaline environments existed [35] and allowed survival of an euryhaline-adapted fauna [36]. Recently, genetic studies using 16s rRNA [37] and cytochrome c oxidase I (COI) [38] sequences further confirmed the distinction of Atlantic and Mediterranean green crabs by showing reciprocal monophyly and a clear genetic break with a 2.5% and a 11% sequence divergence, respectively.

Using a data set of 596 mitochondrial COI sequences of C. *aestuarii* and *C. maenas*, corresponding to 27 sampling locations, we inferred the demographic histories for all differentiated groups within species. Our approach consisted in obtaining an accurate rate calibration by incorporating deep calibration points derived from biogeographic events together with appropriate demographic priors. Results are contrasted with those obtained by a simplified approach using phylogenetic priors that ignores the inter-specific nature of the data and the effect of demographic processes [39]–[41]. Chronologies of past events change significantly with the application of revised molecular rates that, following Ho *et al.* [42], consider a separate demographic model of exponential growth for each individual species. Collectively, our results stress the importance of using adequate calibrations and demographic priors in order to infer more accurately recent evolutionary events, and importantly, to avoid considerable overestimates of evolutionary time scales.

## Results

Diversity indices are summarized in Table 1. A total of 172 haplotypes were recovered among the 596 individuals analyzed, 105 haplotypes in *C. aestuarii* and 67 haplotypes in *C. maenas*. A 10.35% sequence divergence was observed between *C. aestuarii* and *C. maenas*, separated by at least 39 substitutions, all of which were synonymous. Similar values of haplotype (*C. aestuarii*: 0.883; *C. maenas*: 0.858) and nucleotide (*C. aestuarii*: 0.007; *C. maenas*: 0.006) diversity were found across species. Within *C. aestuarii*, West Mediterranean locations showed lower haplotype diversity (0.51–0.64) in comparison with the rest of locations (0.83–0.99), although sample size in one of the two western Mediterranean localities (Banyuls-sur-Mer) was only 17 individuals. All locations showed similar nucleotide diversity (0.002–0.007) except Naples (0.021). Within *C. maenas*, diversities were similar across locations with N>5 individuals sampled (Table 1).

**Table 1:** Diversity indices within *C. aestuarii* and *C. maenas*.

Species	Region	Location	Code	N	H	h	π
*C. aestuarii*	Adriatic-Ionian	Marano, Italy	MA	32	18(11)	0.897	0.005
		Venice, Italy	VE	32	16(5)	0.909	0.004
		Goro, Italy	GO	32	27(18)	0.988	0.007
		Ravenna, Italy	RA	31	23(12)	0.938	0.005
		Lesina, Italy	FG	32	19(12)	0.911	0.005
		Aquatina, Italy	LE	32	15(6)	0.869	0.004
		Taranto, Italy	TA	32	14(7)	0.825	0.004
	Tyrrhenian	Naples, Italy	NAP	29	13(8)	0.892	0.021
	West Mediterranean	Cagliari, Italy	CAG	32	11(6)	0.643	0.006
		Banyuls-sur-Mer, France	BAN	17	5(1)	0.507	0.002
*C. maenas*	Off-shelf Europe	Seltjarnarnes, Iceland	ICE	18	1(0)	0.000	0.000
		Torshavn, Faeroe Islands	TOR	19	2(1)	0.515	0.003
	Northern Europe	Trondheim, Norway	TRO	3	3(0)	1.000	0.007
		Mongstadt, Norway	MON	22	12(5)	0.853	0.004
		Oslo, Norway	OSL	9	5(1)	0.806	0.007
		Goteborg, Sweden	GOT	15	10(3)	0.933	0.007
		Helgoland, Germany	HEL	5	2(0)	0.400	0.001
		Bremerhaven, Germany	BRE	15	7(2)	0.827	0.005
		Hoek van Holland, The Netherlands	HOE	19	7(3)	0.813	0.004
		Den Helder, The Netherlands	NET	45	17(8)	0.784	0.004
	Western Europe	Fowey, England	FOW	14	8(3)	0.890	0.005
		Isle of Wight, England	WIG	3	3(3)	1.000	0.011
		Bilbao, Spain	BIL	15	6(3)	0.648	0.003
		Betanzos, Spain	BET	14	7(4)	0.758	0.003
		Aveiro, Portugal	AVE	23	9(5)	0.795	0.004
		Cadiz, Spain	CAD	47	21(14)	0.864	0.004
		Palmones, Spain	PAL	9	8(2)	0.972	0.005

Reported are: sampling locations, number of individuals (N), number of haplotypes (H, in brackets are exclusive haplotypes), haplotype (h) and nucleotide (π) diversity.

The McDonald-Kreitman test revealed no signatures of positive selection (p = 1.000) after observing all fixed differences between species being synonymous (10 out of 10) and most polymorphic mutations being synonymous as well (111 out of 113). Only 2 out of 113 polymorphic mutations were replacements (non-synonymous). In addition, a codon-based Z-test of selection showed no excess of amino acid replacements between any pair of sequences (p = 1.000 for all pairwise comparisons). Using the program HyPhy, no codons were suggested to be under positive selection as all codons showed higher or equal number of synonymous than non-synonymous substitutions. Collectively, all tests agreed in showing no excess of amino acid replacements, with >98% of changes being silent (synonymous), which is suggestive of the action of purifying selection but not positive selection.

**Figure 2:**
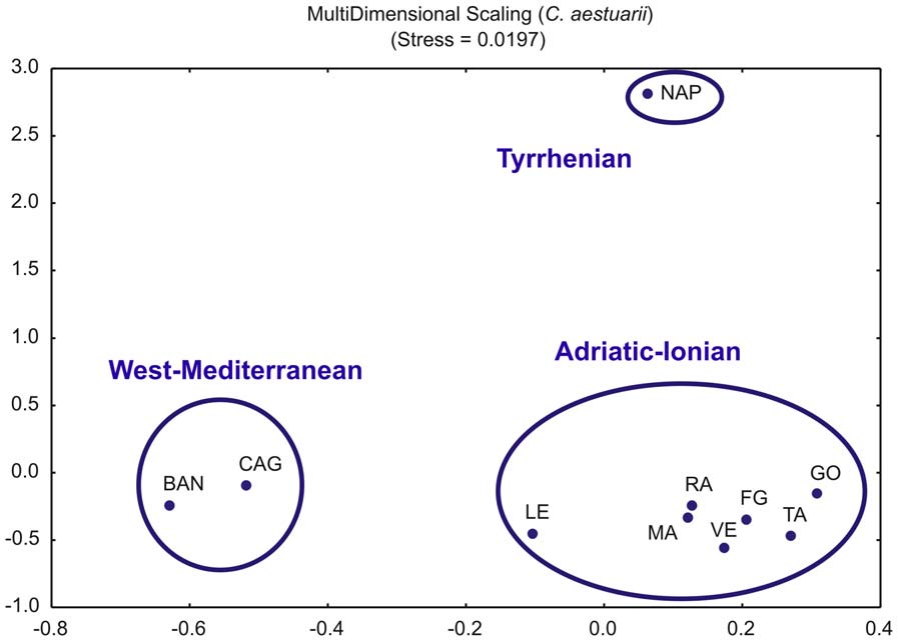
MultiDimensional Scaling analysis (MDS). Plots from MDS analysis for all *C. aestuarii* locations based on pairwise linearized genetic distance (*F*_ST_/(1- *F*_ST_)) values are shown. Samples are labelled as in Table 1. Groupings have been circled for clarity.

Overall genetic differentiation among *C. aestuarii* locations was significant (*F*_ST_ = 0.285, p<0.001). A MultiDimensional Scaling analysis for all *C. aestuarii* locations suggested three different groupings within the Mediterranean Sea: Adriatic-Ionian, Tyrrhenian (Naples) and West Mediterranean (Cagliari, Banyuls-sur-Mer) (Fig. 2). Accordingly, a hierarchical AMOVA partitioned significantly among groups when considering 3 groups (Adriatic-Ionian, Tyrrhenian, West Mediterranean; *F*_CT_ = 0.292, p = 0.002), while no significant differentiation was found among locations within groups (Table 2). Similarly, a significant genetic differentiation was found among *C. maenas* locations (*F*_ST_ = 0.492, p<0.001). Genetic differentiation partitioned significantly when considering the three groups previously proposed by Roman and Palumbi [38], Off-shelf, Northern Europe and Western Europe (*F*_CT_ = 0.470, p<0.001) and was about 10 times larger than the differentiation observed among locations within groups (Table 2).

**Table 2:** Analysis of Molecular Variance (AMOVA).

AMOVA	Sum of squares	Variance	F statistic
***C. aestuarii***			
Among groups	77.77	0.61	*F*_CT_ = 0.292 (p = 0.002)*
Among locations within groups	7.51	−0.01	*F*_SC_ = −0.009 (p = 0.553)
Within locations	431.48	1.48	
(Total)	516.75	2.07	*F*_ST_ = 0.285 (p<0.001)*
***C. maenas***			
Among groups	161.46	0.89	*F*_CT_ = 0.470 (p<0.001)*
Among locations within groups	22.91	0.04	*F*_SC_ = 0.042 (p = 0.004)*
Within locations	267.16	0.96	
(Total)	451.54	1.89	*F*_ST_ = 0.492 (p<0.001)*

Results from partitioning genetic differentiation among groups (*C. aestuarii*: Adriatic/Ionian, Tyrrhenian, Western Mediterranean; *C. maenas*: Off-shelf, Northern Europe, Western Europe) and among populations within groups.

*p<0.005.

**Figure 3:**
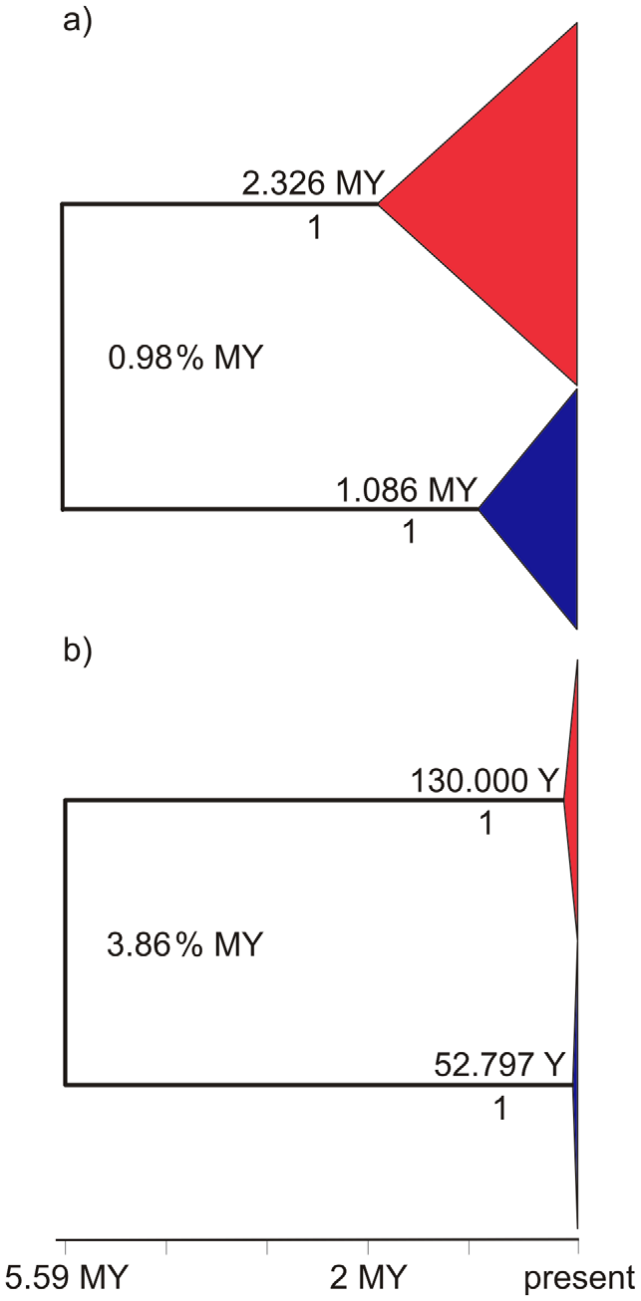
Consensus Trees of the *Carcinus* genus. Trees showing inferred rates and times of the most recent common ancestor (tMRCA) of *C. aestuarii* (red) and *C. maenas* (blue) assuming a total tree height of 5.59 MY. tMRCAs are reported above the branches, and posterior support for the corresponding node, below. (a) Estimates obtained using phylogenetic priors and only unique haplotypes, and (b) Using demographic priors and all sequences.

The evolutionary histories of *C. aestuarii* and *C. maenas* were explored by Bayesian inference. Prior to the analysis, the most appropriate model of sequence evolution was selected using MODELTEST, which suggested an HKY+G substitution model. In a first analysis including only unique haplotypes, using a 5.59 MY split between *C. aestuarii* and *C. maenas* [34] and assuming a Birth-Death prior and a strict clock, two highly supported clades corresponding to the two species were evidenced, with an estimated substitution rate of 1.06% per MY. When repeating the analysis using a relaxed clock, the standard deviation of the uncorrelated lognormal relaxed clock was significantly different than zero (ucld.stdev = 0.26, 95% HPD: 1.06×10^−4^–0.49), indicative of variation in rates among branches and suggesting that a strict clock is not appropriate for our data. Using a relaxed clock, we estimated a substitution rate of 0.98% per MY (Fig. 3), which is congruent with the substitution rate of 0.70–1.15% per MY estimated for crustaceans [43], [44]. The tMRCA was estimated to 2.33 MY (95% HPD: 1.32–3.54 MY) for *C. aestuarii* and 1.09 MY (95% HPD: 0.58–1.67 MY) for *C. maenas*.

When demographic priors were incorporated in the Bayesian analysis by imposing a different exponential prior for each species and including all sequences, the rate of exponential growth was positive for both species, with an associated 95% HPD interval excluding zero (*C. aestuarii*: g = 1.32×10^−4^ years^−1^; 95% HPD: 7.23×10^−6^−4.20x10^−4^ years^−1^; *C. maenas*: g = 3.93×10^−4^ years^−1^; 95% HPD: 1.78×10^−5^−1.28×10^−3^ years^−1^). Bayes factors indicated a much better fit for the model that incorporated demographic priors and allowed exponential growth (ln = −11,345.4) than for a model in which no growth was assumed (ln = −12,457.6), providing a very strong support for the use of exponential demographic priors (2 ln(B_10_)  = 2224.4; [45]). The application of exponential demographic priors resulted in a different tree shape, compressed near the tips and a four-fold higher substitution rate of 3.86% per MY was obtained (Fig. 3). Accordingly, estimated times of the MRCA were younger than before, 130,000 years (95% HPD: 4,595–350,120 years) for *C. aestuarii* and 52,797 years (95% HPD: 2,119–146,500 years) for *C. maenas*. At the intraspecific level, Iceland and Faeroe Islands were characterized exclusively by private haplotypes that clustered in a single group with high posterior support, with an estimate of the time of split between the Off-shelf and continental haplotypes of 45,414 years (95% HPD: 1,764–128,050 years).

Finally, demographic analyses were conducted separately for all groups identified by AMOVA. A pattern of demographic expansion was suggested for the Adriatic-Ionian group in *C. aestuarii* and for the Northern Europe and Western Europe groups in *C. maenas*, all of which were characterized by growth values significantly higher than zero (Table 3). The remaining groups, Tyrrhenian and West Mediterranean in *C. aestuarii* and Off-shelf Europe in *C. maenas*, showed evidence of demographic stability, with HPD values centred around zero. Figure 1 depicts the estimated demographic history of all groups using Bayesian skyline plots. The expansion suggested for the Adriatic-Ionian group in *C. aestuarii* and for the Northern Europe and Western Europe groups in *C. maenas* occurred roughly between 10,000 and 20,000 years ago. The observation of significant negative values of Tajima's D and Fu's FS tests together with Ramos-Onsis and Rozas' R2 values close to zero supports the expansion pattern in the Adriatic-Ionian region in the Mediterranean and along the Atlantic coast (Table 3).

**Table 3:** Demographic analyses.

Location	*g*	95% HPD of *g*		T-D	F-FS	R2
***C. aestuarii***						
Adriatic-Ionian	1.64×10^−4^	9.14×10^−5^	2.46×10^−4^	−2.33***	−26.89***	0.02***
Tyrrhenian	1.93×10^−6^	−4.82×10^−6^	8.67×10^−6^	1.02	0.89	0.16
West Mediterranean	−7.22×10^−7^	−8.04×10^−6^	6.17×10^−6^	−1.98***	−8.48***	0.04*
***C. maenas***						
Off-shelf	−8.49×10^−6^	−4.82×10^−4^	5.76×10^−4^	1.01	3.73	0.17
Northern Europe	1.58×10^−4^	3.63×10^−5^	3.22×10^−4^	−1.77*	−27.02***	0.04*
Western Europe	3.20×10^−4^	1.28×10^−4^	5.55×10^−4^	−2.20***	−27.52***	0.002**

Rate of exponential growth *g* using demographic analyses in BEAST for all *C. aestuarii* and *C. maenas* groups, including 95% highest posterior density interval (HPD), plus Tajima's D statistic (T-D), Fu's FS statistic (F-FS) and Ramos-Onsis and Rozas' R2.

*p<0.05;

**<0.01;

***<0.005.

## Discussion

### Inferring the Demographic Histories of C. aestuarii and C. maenas

Our molecular analysis using COI sequences clearly confirms *C. aestuarii* and *C. maenas* as two separate species in accordance with previous studies [28], [29], [37], [38], with completely supported reciprocal monophyly between haplotypes of the two forms achieved in this extended dataset. An overall sequence divergence of 10.35% has been found, closely matching previous estimates based on a much smaller sampling effort for *C. aestuarii* [38] and also fitting the range described for other crustacean species (9–25%; [46]).

Although the deepest split occurs between the Mediterranean and Atlantic forms, regional genetic substructuring was observed among coastal populations of both *C. aestuarii* and *C. maenas*. MultiDimensional Scaling and AMOVA analyses suggested the existence of three groupings for *C. aestuarii* (Adriatic-Ionian, Tyrrhenian and West Mediterranean Sea). The level of subdivision found might be a consequence of the physical oceanography of the Mediterranean Sea, including (i) circulation being forced by a particularly complex topography limiting water exchange through the various straits, (ii) internal counterclockwise circulation pattern favoring larval retention and (iii) locally deep convection formation acting as barrier between water basins [47]. Recently, Borrero-Pérez *et al*. [48] argued that the major genetic breaks within the Mediterranean are related to the hydrographic isolation of the Adriatic, Aegean and/or Black Sea, after reporting a significant genetic differentiation between Eastern and Western Mediterranean populations of the sea cucumber *Holothuria mammata* using mitochondrial 16S and COI gene sequences. In the case of *C. maenas*, three groupings were also observed (Off-shelf, Northern and Western Europe) in agreement with the previous studies of Roman and Palumbi [38] and Darling *et al.* [28]. The slight but significant break between Western and Northern Europe could reflect regional current patterns, in particular, larval retention could be associated with seasonal circulation in the North Sea [49], [50]. The highest genetic differentiation for *C. maenas* was observed between the off-shelf (Iceland and Faeroe Islands) and the continental-shelf populations. The deep genetic division found is due to the presence of unique haplotypes in the off-shelf region, and might be attributable to the deep water barrier to dispersal in the open sea between the off-shelf and the continental shelf, a pattern suggested for other marine organisms in the region including Atlantic halibut *Hippoglossus hippoglossus* [51], Atlantic herring *Clupea harengus* [52] and plaice *Pleuronectes platessa* [53].

A clear signal of expansion was found for both *C. aestuarii* and *C. maenas*. However, re-analysis taking into account the groupings identified by MultiDimensional Scaling and AMOVA inferred demographic expansion for the Adriatic-Ionian (but not Tyrrhenian nor West Mediterranean) in *C. aestuarii*, and for the Northern and Western Europe groups (but not Off-shore Europe) in *C. maenas*. All expansions were dated between 10,000 and 20,000 years ago, which places the expansion events in a time frame following the Last Glacial Maximum (LGM, between 26,500–20,000 years ago; [54]).

During the LGM, the Mediterranean Sea experienced a dramatic sea-level fluctuation and cold (but ice-free) conditions [47], [55]–[58]. Immediately after the LGM, drastic alterations took place within the Mediterranean Sea, including the rising of the Adriatic Sea, which flooded 18,000 years ago [59]. We hypothesize that the Adriatic Sea could have been colonized by populations of *C. aestuarii* sometime during the last 18,000 years by Mediterranean populations that probably originated from the adjacent Ionian Sea. A group of individuals colonizing a new geographic region and establishing a new population would explain the signature of demographic expansion suggested for the Adriatic group using Bayesian inference. Similarly, a recent study on the demographic history of the marine bivalve *Flexopecten glaber*, suggested the colonization of the Adriatic Sea following the flooding of the Adriatic Sea [60]. The lack of barriers to gene flow between Adriatic and Ionian populations has been demonstrated in many marine species including the sand smelt *Atherina boyeri* [61], [62], the European sprat *Sprattus sprattus* [63] and the sea urchin *Paracentrotus lividus* [64]. The existence of gene exchange between Adriatic-Ionian Sea is evident in our dataset as the Taranto sample from the Ionian Sea clusters together with all the Adriatic samples in the MultiDimensional Scaling analysis. The fact that a demographic expansion in the Mediterranean was only suggested for the Adriatic Sea but not for the Tyrrhenian and West Mediterranean might be due to the lack of new geographic regions to be colonized following the LGM in the western Mediterranean basin. While the flooding of the Adriatic Sea permitted adjacent populations to expand by occupying new habitats, populations from the western Mediterranean did not encounter a similar scenario, which would explain the lack of a demographic expansion signal in those populations.

During the LGM the ice sheet covered most of the North Atlantic from the Barents Sea and the Scandinavian Peninsula to the British Isles [65], [66], with the southern limit of the permafrost located North off the Iberian Peninsula, Italy and the Balkans, which could have acted as refugia [67]. Previous studies have shown that the Quaternary in Europe is characterized by many cycles of contraction of geographic ranges to southern regions during cold periods and expansion northward during subsequent warmings [68]. We propose that during the LGM, *C. maenas* remained in a southern glacial refugium located along the Atlantic coast of the Iberian Peninsula. When the ice sheet retreated after the LGM, *C. maenas* carried out an extensive northward colonization following the European Atlantic coast, recolonizing the newly opened coastal habitats off northern Europe including the Irish Sea, the North Sea and the Baltic Sea, which would explain the signatures of demographic expansion for the Atlantic continental-shelf populations of *C. maenas* in our study. A similar pattern of post-glacial expansion has been suggested for the common goby *Pomatoschistus microps* [69] and the Atlantic cod *Gadus morhua* [70]. Alternatively, additional refugia might have existed in northern Europe (i.e. a glacial lake potentially served as refugium in the southern North Sea; [71]). This could explain the similar genetic diversities found across continental regions in our study, as opposed to the expected high genetic diversity in regions close to the potential refugia (Spain-Portugal) and low genetic diversity in more distant areas.

In contrast with the continental-shelf populations, Bayesian inference suggested a stationary population for the off-shelf region (Faeroe Islands and Iceland), which is congruent with the low genetic diversity found: a single haplotype out of 18 individuals in Iceland and two haplotypes out of 19 individuals in Faeroe Islands, one shared with Iceland. Roman and Palumbi [38] attributed the reduced diversity found in Iceland to a relatively recent colonization event, by means of a stepping-stone range expansion from the northern UK to the Shetland Islands, Faeroe Islands and Iceland after deglaciation. However, our analysis estimates the age of the most recent common ancestor of the off-shore and continental groups to be around 45,000 years, which places it before the LGM [54]. Although the confidence intervals of our dates still incorporate the time period post LGM, we propose that a separate refugium area existed in the off-shelf during the LGM that was the origin of the current Faeroe Islands and Iceland populations, as alternative to a stepping-stone range expansion after the LGM. A potential glacial refugium in Iceland seems plausible on the basis of a suggested limited glaciation, with large areas of northern Iceland remaining ice-free during the LGM [72]. The low genetic diversity observed in this island group might be consequence of a small founder population size, which has remained of constant low size over time (as suggested by the Bayesian analysis) and has not expanded because of the impossibility of finding new habitats due to its isolation. The geographic position of the Faeroe Islands and Iceland at the northern limit of the geographical range of *C. maenas* might also account for the low genetic variation, which is expected in marginal populations at the edges of species distribution range [73]. The existence of two separate refugia is supported by a Bayes factor analysis in which a model with one refugium only (with no constrain on the topology of the tree) was compared with a two refugia model (imposing the monophyly of the two clades, Iceland and the rest of Europe). Twice the difference in logarithm of harmonic means of likelihoods was compared using the approach of Kass and Raftery [45]. The two refugia model fits the data better, with a 2 ln(B_10_) of 6.412, suggesting a strong evidence against the one refugium model.

### Appropriate Calibration using Demographic Information

By demonstrating that estimated divergence times within species change significantly when including prior information on demographic processes, our findings reiterate the importance of using appropriate calibrations in analyses of recent evolutionary events.

Ho *et al.* [25] advocated the use of internal, intraspecific calibration points as opposed to using deep calibration points, particularly those based on the fossil record. Alternatively, we demonstrate that a more accurate rate calibration can be obtained by using appropriate demographic priors together with deep calibration points obtained from biogeography. Our study shows that the application of revised molecular rates that consider separate demographic models of exponential growth for each species allows the proper inference of chronologies within species. Our study also denotes a strong underestimation of substitution rates when ignoring evidence for recent population expansion. In the case of *C. aestuarii* and *C. maenas*, a four-fold higher rate (3.86% per MY) was obtained when demographic information was incorporated into the analysis by means of imposing an exponential prior for each species. Bayes factors provided a very strong evidence against a model with no growth, supporting the use of exponential demographic priors. This rate strongly differs from the commonly used crustacean rate of 0.70–1.15% per MY [43], [44] and from the rate inferred by the simplified phylogenetic approach in our study (0.98% per MY). The latter differs only slightly from a rate of 0.93% MY that can be inferred considering the uncorrected divergence between species (10.35%) and our calibration point (5.59 MY), which indicates that in our case the inclusion of a relaxed clock and the consideration of best fit model of sequence evolution has only a minor impact on rate estimates.

Under the time dependency of molecular rate estimates hypothesis, the rate heterogeneity at different scales is associated with the fact that slightly deleterious mutations contribute to measured genetic differences at the shallow time scale but are progressively removed by purifying selection during further evolutionary divergence [15], [17], [19], [26], [74]. In our dataset, tests of selection showed >98% of all changes to be synonymous, with no excess of amino acid replacements, which is suggestive of the action of purifying selection. Importantly, our study provides evidence that recent demographic history can play a role in producing a much higher level of genetic polymorphism at the intraspecific level that results in higher rates. In this sense, demography and purifying selection might be acting in concert considering that in recently expanded populations slightly deleterious mutations can accumulate in the gene pool due to the large population sizes [75].

Typically, many published studies on historical demography are based on the identification of population expansions using neutrality tests (Tajima's D, Fu's FS, Ramos-Onsis and Rozas' R2) or the estimation of time of sudden expansion by mismatch distribution and application of universal clock rates [76]–[79]. Those studies ignore the effects of rate calibration, model of sequence evolution and population history, which might result in an overestimation of evolutionary time scales. Furthermore, the advent of Bayesian methods to reconstruct historical demographies leaves little space for the use of simplified methods such as neutrality tests or mismatch distribution that cannot account for rate heterogeneity and are based on expectations of simple models of sudden population expansion. As an example, our study provides insights on the use of neutrality tests to pick up a signal of population expansion. All neutrality tests congruently identified a demographic expansion in the Adriatic, Northern and Western Europe and identified the Tyrrhenian and Atlantic Off-shore as stationary populations. However, all neutrality tests suggested a strong expansion for the West Mediterranean, which was not suggested by BEAST nor supported by the paleoclimatic history of this region. In view of our results, neutrality tests should be complemented by approaches based on more complex demographic models, in spite of increased computational time.

In summary, our findings point out the difficulties in producing accurate date estimates due to rate heterogeneity when recent calibration points are not available. The approach proposed in our study can be summarized in the following steps: (i) use calibration points based on biogeographic events or fossil records if the former are not available; (ii) use all individuals for the analysis, not only unique haplotypes; (iii) incorporate accurate demographic priors, allowing for exponential growth when suggested; (iv) estimate dates in a relaxed clock framework that allows for rate variation among branches; and (v) perform analyses for all data and separately for all differentiated groups within species as identified by AMOVA analyses. Our data clearly show that analyses performed within this framework represent an improved method for calibrating estimates of divergence times and evolutionary histories from molecular data. Finally, it should be stressed that one limitation of our study is the use of a single mitochondrial gene (reflecting only the maternal line and possibly insensitive to population size [80]) without confirmation from nuclear genes. Complementary data based on other mitochondrial or nuclear data could be useful to compare maternally-inherited and biparental markers and to detect how male-mediated gene flow might have influenced the global evolutionary history of the species.

## Materials and Methods

Samples of *C. aestuarii* (N = 255) were collected during 2005–2008 in 8 locations in the Mediterranean Sea (Table 1, Figure 1). Specimens were frozen at −80°C after collection. Total genomic DNA was extracted from chelipeds using a salting out protocol [81]. We amplified a 569 base-pair (bp) fragment of the mitochondrial COI (cytochrome oxidase subunit I) gene using primers developed by Roman and Palumbi [38] for *C. maenas*. PCR products were obtained in a GeneAmp PCR System 2700 Thermocycler (Applied Biosystems). PCR reactions and conditions followed Roman and Palumbi (2004) and PCR products were sequenced using an ABI 3130 AVANT automatic capillary sequencer (Applied Biosystems) on both strands. All sequences have been deposited on GenBank (full details are given in Table S1). Additionally, we analyzed 46 COI sequences from two Mediterranean locations of *C. aestuarii*, Banyuls-sur-Mer and Naples, and 295 COI sequences of *C. maenas* obtained from GenBank (accession numbers FJ159008 - FJ159102; [28]). In total, the data set comprised 596 individuals corresponding to 27 sampling locations, 10 Mediterranean and 17 Atlantic (Table 1).

Mitochondrial DNA sequences were aligned using Clustal_X [82] and 480 bp of overlapping sequences were used for all subsequent analyses. We used DnaSP v.5 [83] to calculate haplotype (h) and nucleotide (π) diversity, McDonald-Kreitman's [84] test of selection, Tajima's [85] D statistic test, Fu's [86] F statistic and Ramos-Onsis and Rozas' [87] R2 statistic, together with the uncorrected sequence divergence between species. We used MEGA 5 [88] to conduct the codon-based Z-test of selection and test for selection across codons using HyPhy.

Genetic differentiation was explored by calculating pairwise *F*_ST_ values between all locations. Significance tests were assessed with 10,000 permutations. A multivariate ordination was conducted by MultiDimensional Scaling analysis using STATISTICA (StatSoft). Non-hierarchical and hierarchical analysis of molecular variance (AMOVA; [89]) using ARLEQUIN [90] was conducted to explore the relative contribution of molecular variance at different geographical scales (localities included in each group are reported in Table 1). Uncorrected pairwise differences were used to partition molecular variance among regions and among sampling locations within regions.

The demographic histories of *C. aestuarii* and *C. maenas* were estimated using a Bayesian Markov-Chain Monte Carlo (MCMC) coalescent approach implemented in BEAST 1.4.8 [13]. Prior to the analysis, the most appropriate model of sequence evolution for the data set was selected using MODELTEST v3.06 [91] based on Akaike Information Criterion scores. The closure of the Gibraltar strait at the start of the Messinian Salinity Crisis (5.59 MY ago; [34]) was used as calibration point for rate estimation. During the Messinian Salinity Crisis, the contact between the Mediterranean and the Atlantic Ocean was interrupted altogether, thus providing the geographic barrier necessary to the speciation of *C. aestuarii* and *C. maenas* [33]. Uncertainty on the divergence time was modeled using a normal prior with a standard deviation of 55,000 years.

In the first step, a simplified phylogenetic approach was used, ignoring the intraspecific nature of our data, using a Birth-Death prior [92], [93] and including only unique haplotype sequences. Analyses were repeated with a strict clock and an uncorrelated lognormal relaxed clock [9]. For each model, the MCMC was run for 100 million steps and sampled every 1,000 steps. The first 10% steps of each run were discarded as burn-in, and after checking convergence by examining Effective Sample Sizes (ESS) of all parameters using Tracer v1.5 [94], molecular rate and time of the most recent common ancestor (tMRCA) for the two species were determined and reported as mean value with 95% highest posterior density interval (HPD).

In the second step, we followed the approach of Ho *et al.* [42], which consisted of applying a separate demographic model of exponential growth to each species in the form of a coalescent prior. All sequences were used, not merely unique haplotypes. Considering the size of the dataset (N = 596), exponential growth was chosen to avoid over parameterization of the model, maintaining the possibility to estimate population growth, decline or constant size. The exponential growth prior was restricted to ingroups and a normal prior on the age of the root was placed as before. Following previous analyses, an uncorrelated relaxed clock was used to accommodate rate variation among lineages. The MCMC was run as described above but the sampling was performed each 100,000 steps and the analysis was repeated (N = 8) until acceptable mixing (ESS>100) and convergence to the stationary distribution were recovered. Samples from different runs were combined and model parameters estimated. In addition to overall substitution rate and tMRCAs, growth rate was also estimated. Bayes factors, calculated as the harmonic mean of likelihoods [95] were used to evaluate the adequacy of the exponential growth model, following Kass & Raftery [45].

In the final step, demographic Bayesian analyses were performed separately for all differentiated groups within species (as identified by AMOVA analyses), including all sequences of a given group and importing the rate estimated in the previous steps as normal prior with appropriate mean and standard deviation. Demographic histories were estimated using both an exponential prior and a Bayesian skyline plot setting the number of groups to m = 5 and the parameterization described above. MCMC was run for 50 million steps with sampling performed every 10,000 steps.

## Supporting Information

Table S1 **GenBank accession numbers and distribution in the populations for** ***C. aestuarii*** **COI haplotypes obtained in this study.**
(DOC)
