# Supplementary material for: Reconciling Deep Calibration and Demographic History: Bayesian Inference of Post Glacial Colonization Patterns in Carcinus aestuarii (Nardo, 1847) and C. maenas (Linnaeus, 1758)
Source: PLoS One. 2011 Dec 2;6(12):e28567. doi: 10.1371/journal.pone.0028567 (PMC3229605; doi:10.1371/journal.pone.0028567)
Supplement: Table S1 — GenBank accession numbers and distribution in the populations for C. aestuarii COI haplotypes obtained in this study. (DOC) [file pone.0028567.s001.doc]

**Table S1.** GenBank accession numbers and distribution in the populations for *C. aestuarii* COI haplotypes obtained in this study.

| **Haplotype** | **Accession number** | **MA** | **VE** | **GO** | **RA** | **FG** | **LE** | **TA** | **CAG** | **TOTAL** |
| --- | --- | --- | --- | --- | --- | --- | --- | --- | --- | --- |
| CA04 | JN989973 | 10 | 8 | 2 | 8 | 9 | 11 | 13 | 19 | 80 |
| CA13 | JN989974 |  | 1 |  |  | 1 |  |  |  | 2 |
| CA23 | JN989975 | 2 | 2 |  | 1 | 1 |  |  |  | 6 |
| CAG06 | JN989976 |  |  |  |  |  |  |  | 1 | 1 |
| CAG08 | JN989977 |  |  |  | 1 |  |  |  | 4 | 5 |
| CAG11 | JN989978 |  |  | 1 | 1 |  |  |  | 1 | 3 |
| CAG24 | JN989979 |  |  |  |  |  |  |  | 1 | 1 |
| CAG26 | JN989980 |  |  |  |  |  |  |  | 1 | 1 |
| CAG33 | JN989981 |  |  |  |  |  |  |  | 1 | 1 |
| CAG34 | JN989982 |  |  |  |  |  |  |  | 1 | 1 |
| CAG44 | JN989983 |  |  |  |  |  |  |  | 1 | 1 |
| CE110 | JN989984 |  | 1 |  |  |  |  |  |  | 1 |
| CE132 | JN989985 |  | 1 |  |  |  |  |  |  | 1 |
| CI105 | JN989986 | 1 | 3 | 3 | 1 | 2 | 3 | 4 |  | 17 |
| CZ33 | JN989987 |  | 1 |  |  |  |  |  |  | 1 |
| FG03 | JN989988 |  |  |  |  | 1 |  |  |  | 1 |
| FG11 | JN989989 |  |  |  |  | 1 |  |  |  | 1 |
| FG14 | JN989990 |  |  |  |  | 1 |  |  |  | 1 |
| FG16 | JN989991 |  |  |  |  | 1 |  |  |  | 1 |
| FG17 | JN989992 |  |  |  |  | 1 |  |  |  | 1 |
| FG18 | JN989993 |  |  |  |  | 1 |  |  |  | 1 |
| FG19 | JN989994 |  |  |  |  | 1 |  |  |  | 1 |
| FG20 | JN989995 |  |  |  |  | 1 |  |  |  | 1 |
| FG25 | JN989996 | 2 |  | 2 | 1 | 2 |  |  |  | 7 |
| FG33 | JN989997 |  |  |  |  | 1 |  |  |  | 1 |
| FG37 | JN989998 |  |  |  |  | 1 |  |  |  | 1 |
| FG38 | JN989999 |  |  |  |  | 1 |  |  |  | 1 |
| FG40 | JN990000 |  |  |  |  | 1 |  |  |  | 1 |
| FU110 | JN990001 | 3 | 5 | 2 | 2 | 4 | 3 | 2 |  | 21 |
| FU126 | JN990002 |  | 1 |  |  |  | 2 |  |  | 3 |
| FU144 | JN990003 |  | 1 |  |  |  |  |  |  | 1 |
| GO02 | JN990004 |  |  | 1 |  |  |  |  |  | 1 |
| GO03 | JN990005 |  |  | 1 |  |  |  |  |  | 1 |
| GO04 | JN990006 |  |  | 1 |  |  |  |  |  | 1 |
| GO10 | JN990007 |  |  | 1 |  |  |  |  |  | 1 |
| GO15 | JN990008 |  |  | 1 |  |  |  |  |  | 1 |
| GO16 | JN990009 |  |  | 1 |  |  |  |  |  | 1 |
| GO17 | JN990010 |  |  | 1 |  |  |  |  |  | 1 |
| GO18 | JN990011 |  |  | 1 |  |  |  |  |  | 1 |
| GO19 | JN990012 |  |  | 1 |  |  |  |  |  | 1 |
| GO25 | JN990013 |  |  | 1 |  |  |  |  |  | 1 |
| GO27 | JN990014 |  |  | 1 |  |  |  |  |  | 1 |
| GO28 | JN990015 |  |  | 1 |  |  |  |  |  | 1 |
| GO29 | JN990016 |  |  | 1 |  |  | 1 |  |  | 2 |
| GO30 | JN990017 |  |  | 1 |  |  |  |  |  | 1 |
| GO31 | JN990018 |  |  | 1 |  |  | 1 |  |  | 2 |
| GO32 | JN990019 |  |  | 1 |  |  |  |  |  | 1 |
| GO35 | JN990020 |  |  | 1 |  |  |  |  |  | 1 |
| GO47 | JN990021 |  |  | 1 |  |  |  |  |  | 1 |
| GO56 | JN990022 |  | 1 | 1 | 1 |  |  | 1 |  | 4 |
| GO57 | JN990023 |  |  | 1 |  |  |  |  |  | 1 |
| GO58 | JN990024 |  |  | 1 |  |  |  |  |  | 1 |
| LE04 | JN990025 |  |  |  |  |  | 1 |  |  | 1 |
| LE24 | JN990026 |  |  |  |  |  | 3 | 1 |  | 4 |
| LE32 | JN990027 |  |  |  |  |  | 1 |  |  | 1 |
| LE47 | JN990028 |  |  |  |  |  | 1 |  |  | 1 |
| LE49 | JN990029 |  |  |  |  |  | 1 |  |  | 1 |
| LE50 | JN990030 | 1 | 1 |  | 1 |  | 1 |  |  | 4 |
| LE53 | JN990031 |  |  |  |  |  | 1 |  |  | 1 |
| LE54 | JN990032 |  |  |  |  |  | 1 |  |  | 1 |
| MA02 | JN990033 | 1 |  |  | 1 |  |  |  |  | 2 |
| MA04 | JN990034 | 1 |  |  |  |  |  |  |  | 1 |
| MA05 | JN990035 | 1 |  |  |  |  |  |  |  | 1 |
| MA08 | JN990036 | 1 |  |  |  |  |  |  |  | 1 |
| MA09 | JN990037 | 1 |  |  |  |  |  |  |  | 1 |
| MA10 | JN990038 | 2 |  |  |  |  |  |  |  | 2 |
| MA11 | JN990039 | 1 |  |  |  |  |  |  |  | 1 |
| MA14 | JN990040 | 1 |  |  |  |  |  |  |  | 1 |
| MA21 | JN990041 | 1 |  |  |  |  |  |  |  | 1 |
| MA23 | JN990042 | 1 |  |  |  |  |  |  |  | 1 |
| MA30 | JN990043 | 1 |  |  |  |  |  |  |  | 1 |
| MA54 | JN990044 | 1 |  |  |  |  |  |  |  | 1 |
| PC16 | JN990045 |  | 1 |  |  |  |  |  |  | 1 |
| RA08 | JN990046 |  |  |  | 1 |  |  | 1 |  | 2 |
| RA10 | JN990047 |  |  |  | 1 |  |  |  |  | 1 |
| RA12 | JN990048 |  |  |  | 1 |  |  |  |  | 1 |
| RA16 | JN990049 |  |  |  | 1 |  |  |  |  | 1 |
| RA17 | JN990050 |  |  |  | 1 |  |  |  |  | 1 |
| RA30 | JN990051 |  |  |  | 1 |  |  |  |  | 1 |
| RA32 | JN990052 |  |  |  | 1 |  |  |  |  | 1 |
| RA33 | JN990053 |  |  |  | 1 |  |  |  |  | 1 |
| RA34 | JN990054 |  |  |  | 1 |  |  |  |  | 1 |
| RA38 | JN990055 |  |  |  | 1 |  |  |  |  | 1 |
| RA45 | JN990056 |  |  |  | 1 |  |  |  |  | 1 |
| RA46 | JN990057 |  |  |  | 1 |  |  |  |  | 1 |
| RA49 | JN990058 |  |  |  | 1 |  |  |  |  | 1 |
| SAL27 | JN990059 |  | 1 | 1 |  |  |  |  |  | 2 |
| SC20 | JN990060 |  | 3 |  |  | 1 | 1 |  | 1 | 6 |
| TA05 | JN990061 |  |  |  |  |  |  | 2 |  | 2 |
| TA07 | JN990062 |  |  |  |  |  |  | 2 |  | 2 |
| TA10 | JN990063 |  |  |  |  |  |  | 1 |  | 1 |
| TA20 | JN990064 |  |  |  |  |  |  | 1 |  | 1 |
| TA21 | JN990065 |  |  |  |  |  |  | 1 |  | 1 |
| TA22 | JN990066 |  |  |  |  |  |  | 1 |  | 1 |
| TA23 | JN990067 |  | 1 |  |  |  |  | 1 | 1 | 3 |
| TA31 | JN990068 |  |  |  |  |  |  | 1 |  | 1 |
| **TOTAL** |  | 32 | 32 | 32 | 31 | 32 | 32 | 32 | 32 | 255 |
